# Exercise interventions for older adults with Alzheimer’s disease: a systematic review and meta-analysis protocol

**DOI:** 10.1186/s13643-020-01555-8

**Published:** 2021-01-04

**Authors:** Julie M. Faieta, Hannes Devos, Prasanna Vaduvathiriyan, Michele K. York, Kirk I. Erickson, Mark A. Hirsch, Brian G. Downer, Erwin E. H. van Wegen, Diana C. Wong, Elena Philippou, Ahmed Negm, Pedram Ahmadnezhad, Shilpa Krishnan, Melike Kahya, Pallavi Sood, Patricia C. Heyn

**Affiliations:** 1 Department of Rehabilitation, Université Laval, Quebec, Canada; 2 Centre interdisciplinaire de recherche en réadaptation et en intégration sociale (CIRRIS), Québec, Canada; 3 Department of Physical Therapy and Rehabilitation Science, University of Kansas Medical Center, Kansas City, USA; 4 Dykes library, University of Kansas Medical Center, Kansas City, USA; 5 Neurology, Baylor College of Medicine, Houston, USA; 6 Psychology, University of Pittsburgh, Pittsburgh, USA; 7 Physical Medicine and Rehabilitation, Carolinas Medical Center/Carolinas Rehabilitation, Charlotte, USA; 8 Division of Rehabilitation Sciences, School of Health Professions, University of Texas Medical Branch, Galveston, USA; 9 Department of Rehabilitation Medicine, Amsterdam Neurosciences, Amsterdam Movement Sciences, Amsterdam, Netherlands; 10 Occupational Therapy, Rehabilitation Department, New York University, New York, USA; 11 Life and Health Sciences, University of Nicosia, Nicosia, Cyprus; 12 Rehabilitation Medicine, University of Alberta, Edmonton, Canada; 13 Rehabilitation Medicine, Division of Physical Therapy, Emory University, Atlanta, USA; 14 Rehabilitation Science, University of Florida, Gainesville, USA; 15 Physical Medicine and Rehabilitation, University of Colorado Anschutz Medical Campus, Aurora, USA

**Keywords:** Exercise, Physical activity, Exercise training, Older adults, Dementia, Alzheimer’s disease, Mild cognitive impairment, Cognitive function

## Abstract

**Background:**

The growing societal and economic impact of Alzheimer’s disease (AD) is further compounded by the present lack of disease-modifying interventions. Non-pharmacological intervention approaches, such as exercise, have the potential to be powerful approaches to improve or mitigate the symptoms of AD without added side effects or financial burden associated with drug therapies. Various forms and regiments of exercise (i.e., strength, aerobic, multicomponent) have been reported in the literature; however, conflicting evidence obscures clear interpretation of the value and impact of exercise as an intervention for older adults with AD. The primary objective of this review will be to evaluate the effects of exercise interventions for older adults with AD. In addition, this review will evaluate the evidence quality and synthesize the exercise training prescriptions for proper clinical practice guidelines and recommendations.

**Methods:**

This systematic review and meta-analysis will be carried out by an interdisciplinary collective representing clinical and research stakeholders with diverse expertise related to neurodegenerative diseases and rehabilitation medicine. Literature sources will include the following: Embase, PsychINFO, OVID Medline, and Ovid MEDLINE(R) and Epub Ahead of Print, In-Process & Other Non-Indexed Citations and Daily. Inclusion criteria are participants with late onset AD and structured exercise interventions with prescribed duration, frequency, and intensity. The primary outcome of this study will center on improved or sustained cognitive functioning. Secondary outcomes will include institutionalization-related outcomes, ability in activities of daily living, mood and emotional well-being, quality of life, morbidity, and mortality. Analysis procedures to include measurement of bias, data synthesis, sensitivity analysis, and assessment of heterogeneity are described in this protocol.

**Discussion:**

This review is anticipated to yield clinically meaningful insight on the specific value of exercise for older adults with AD. Improved understanding of diverse exercise intervention approaches and their specific impact on various health- and function-related outcomes is expected to guide clinicians to more frequently and accurately prescribe meaningful interventions for those affected by AD.

**Systematic review registration:**

PROSPERO CRD42020175016.

**Supplementary Information:**

The online version contains supplementary material available at 10.1186/s13643-020-01555-8.

## Background

Characterized by deficits in memory, cognitive function, and behavior [1], dementia is projected to affect over 48 million individuals globally in 2020, and to increase by over 46% to a prevalence of 90.3 million by 2040 [2]. Alzheimer’s disease (AD) is the most prevalent form of dementia [3], accounting for approximately 60–80% of the dementia population [4] and affecting over 5.5 million individuals in the USA alone [5]. Alzheimer’s disease is a progressive, irreversible brain disorder which impacts memory and thinking, and eventually the ability to carry out basic activities of daily living. Typically, clinical symptoms include declines in episodic memory, behavioral changes, and impaired language and visuospatial functioning [6]. Due to the functional changes associated with the disease, many individuals living with AD in the community require the help and support of at least one informal caregiver, either a family member or a friend. The Alzheimer’s Association reports that the extensive care provided by informal caregivers is valued at over *18 billion dollars* annually [4]. Current pharmacotherapy interventions for AD are symptomatic, masking rather than altering the course of the disease [7]. Therefore, non-pharmacological intervention options such as exercise are of great importance in caring for individuals living with the symptoms of AD.

A physically active lifestyle that incorporates exercise, whether through targeted training or recreation, is frequently promoted as a component of a healthy lifestyle. The familiarity of exercise as a wellness concept may lessen its perceived importance. However, exercise should be viewed as a highly impactful tool associated with a myriad of widely accepted benefits such as cardiovascular fitness, improved sleep, and digestive functioning [8]. Specific to healthy aging, research has shown physical activity to delay cognitive decline [9, 10]. Exercise-based interventions can be practically implemented at a community-wide level to support mental health and wellness in the aging populations via recommended guidelines and community initiatives (e.g., SilverSneakers type programs). In addition to general health and wellness, exercise is a valuable tool across health care settings. Exercise is used across various rehabilitative settings to target skeletal muscle function following an injury or to improve overall functional capacity. Additionally, exercise has shown promise as an effective cognitive enhancement intervention for older adults with cognitive impairments [11–14].

The mechanisms through which exercise may benefit cognitive functioning are likely to be multifactorial. Stillman and colleagues indicate that these mechanisms can be conceptualized on multiple levels to include cellular, molecular, and psychosocial levels [15]. On the molecular and cellular level, sustained routine aerobic exercise in adults reduces inflammation, regulates insulin pathways, suppresses oxidative stress, and contributes to calcium homeostasis [16], all of which may independently or additively influence brain and cognitive outcomes. Furthermore, exercise has been shown to promote various plasticity-related events in the human brain including neurogenesis, synaptogenesis, and angiogenesis. Several randomized trials have demonstrated that exercise alters the size of brain regions in older adults, and these changes might be linked to improvements in cognitive performance [17, 18]. Yet, few neuroimaging studies have attempted to links these changes to behavior which inevitably leads to the question of the relevance and importance of brain changes detected using neuroimaging methods. Additional consensus of clinical trials of exercise including both neuroimaging and cognitive outcomes are needed.

Mechanisms of exercise effect on cognitive function can also be conceptualized at the psychosocial level. For example, exercise-induced changes in sleep parameters may be mediating cognitive benefits observed with exercise. Another possibility is that the mood-enhancing effects of exercise lead to cognitive improvements. Although these are all possibilities, they are largely speculation at this point given the dearth of literature that tests such hypotheses. One likely scenario is that there are many different pathways that explain how exercise influences cognition and that these pathways might differ as a function of diverse variables, such as, age group, disease state, mode of exercise, duration and volume of exercise, intensity of exercise, or baseline cognitive status.

Despite an increased number of both systematic reviews and meta-analyses published in the last 15 years on the topic of physical activity and dementia, few have attempted to evaluate whether physical activity or exercise prescription was effective or in support of current clinical practice recommendations by the American College of Sports Medicine (e.g., Exercise is Medicine (EIM) guideline [19]). Furthermore, some findings indicate that exercise is ineffective in delaying cognitive decline [20]. A recent review of the literature noted inconsistency in the evidence on the value of physical activity for cognitive outcomes in the AD population, hypothesizing that changes in standardized outcome measures used to assess cognition may be a contributing factor to this apparent incongruity [21]. Studies that do show effectiveness of exercise intervention demonstrate, at best, moderate effects on cognitive function in AD [22] or simply indicate that evidence is insufficient to draw conclusions [23]. Limited and conflicting evidence thus generates more questions than answers. The long-term impact of effective interventions is unclear, and the type and optimal training parameters (frequency, intensity, and duration) leading to the most effective outcomes have not yet been established. Thus, to address the present lack of understanding, this review aims to develop a more comprehensive analysis of the potential cognitive functioning outcomes related to exercise in individuals with AD. This review will not only evaluate the evidence, its quality, and bias, but it will also synthesize the intervention doses and prescriptions for proper clinical practice guidelines and recommendations. The need to evaluate specific exercise dosages is highlighted by Guitar et al. where the authors concluded that “Future studies should explore the benefits of the American College of Sports Medicine recommended 150 min of physical exercise per week with select measures of executive function [24], p. 159.”

As research presses toward a cure for AD, it is imperative to critically assess the existing, feasible intervention approaches in order to support the quality of life for those already experiencing AD-related decline. Exercise has the potential to mitigate the physical and cognitive decline associated with AD progression, but inconsistent support for exercise as an intervention for cognitive function necessitates critical assessment of the literature. This systematic review protocol details the aims, methods, and anticipated findings of an upcoming review on exercise interventions for older adults with Alzheimer’s disease. The primary objective of the present systematic review and meta-analysis is to assess the effects of structured exercise regiments on cognitive function in older adults with AD. Secondary objectives of this review include assessing the effects of structured exercise regiments on institutionalization, activities of daily living, emotional well-being, quality of life, morbidity, and mortality.

## Methods

This protocol has been registered under the PROSPERO database (registration number CRD42020175016). We will follow the standards for best practice in transparent, reproducible, and ethical reporting of systematic reviews per the Preferred Reporting Items for Systematic Reviews and Meta-Analyses Protocols (PRISMA-P) statement (see Additional File 1) [25].

### Eligibility criteria

For the purposes of this review, only randomized controlled trials will be considered. We will evaluate and include studies according to the following criteria: participants, interventions, outcome(s) of interest, and study design. Participant inclusion criteria will include at least one sample group with Alzheimer’s disease, as described by individual study authors. We will include studies with additional diagnostic or caregiver groups, but inclusion of participants with AD and outcomes specific to those with this condition will be considered an inclusion criterion. To be considered eligible for inclusion, studies must also contain one or more exercise-based intervention. To be able to evaluate the exercise prescriptions (dose, mode, type), we will consider only structured and planned exercise interventions of prescribed frequency, dose, and/or intensity as specified by study authors. For the purposes of this review, we will use the following definition of exercise, sourced from the Physical Activity Guidelines Advisory Committee “physical activity that is planned, structured, repetitive, and designed to improve or maintain physical fitness, physical performance, or health [26]. Exercise, like physical activity, encompasses all intensities [27], p. C-3.”. We will use the term exercise synonymously with structured physical activity throughout this review.

Based on this definition, we will include both resistance and aerobic exercises. Examples include, but are not limited to, walking, swimming, weight training, and use of exercise equipment in a home or gym setting that are reported with prescribed time, frequency, and/or intensity (see examples in Table 1).

**Table 1:** Example intervention parameters

Activity	Frequency	Dose	Intensity
Walking	30 min, 2× per week	10 weeks	Moderate pace
Waltz dancing	1 h, 1× per week	7 weeks	Intermediate dance
Pilates	45 min, 3× per week	5 weeks	Low intensity
Pickle ball	3-h sets, 2× per month	6 months	5 METs

We will classify comparative doses of exercise as uniform with the primary intervention group (for example, strength training compared to alternative dose of strength training) or variant (for example, strength training, compared to non-strength training forms of exercise). Only control (non-exercise groups) and comparative dose groups will be considered.

### Outcomes

The primary outcomes of our review will be cognitive improvement and/or sustained cognitive functioning in older adults with AD. While AD impacts many areas of behavioral and psychosocial functioning, this review seeks to provide an in-depth and comprehensive investigation on a narrow area deeply affected by AD, specifically, the impact of exercise on cognitive ability within AD populations. Therefore, cognitive function will be considered our primary area of focus. We will also explore specific cognitive domains (i.e., memory, language, attention and executive function, and visuospatial function).

Secondary outcomes will be explored through sub-analyses pending review results. While our primary area of concern is cognitive function, we will also consider additional outcomes that are typically associated with AD disease progression to better understand the global impact of exercise on AD. Anticipated secondary outcomes of interest include the impact of exercise on:

- Institutionalization-related outcomes (such as caregiver burden, level of assistance for activities of daily living, time to and rate of institutionalization);
- Ability in functional activities and activities of daily living;
- Mood and emotional well-being;
- Caregiver outcomes such as health, depression, participation, and function;
- Quality of life-related outcomes (such as social participation);
- Morbidity and overall mortality.

Both clinically important and statistically significant differences will be included as appropriate. Finally, we will report applicable incidence of adverse events such as injury or pain with exercise participation.

### Information sources and search strategy

We will conduct comprehensive literature searches in the following databases (from their inception onwards):

- Embase.com, 1947–present
- Ovid Medline, Ovid MEDLINE(R) and Epub Ahead of Print, In-Process & Other Non-Indexed Citations, Daily and Versions(R), 1946 to present.
- PsycINFO (Ebsco interface), 1734–present

A combination of keywords and controlled vocabularies (when available in the databases) are used for conducting the searches. Search techniques such as adjacency search and truncating are used in order to increase the sensitivity in results. Search results will be exported, and duplicates will be removed using EndNote [version X9.2]. The draft search strategies are provided in Additional file 2.

### Screening and selection procedure

All titles and abstracts will be reviewed by two independent reviewers to determine appropriateness to the purposes of this review. A third reviewer will resolve any conflicts through consensus. Similarly, two authors will review full texts independently and compare these against predefined eligibility criteria. If necessary, review authors will contact article authors to confirm the article’s appropriateness for inclusion in this review. All review authors hold expertise relevant to the review subject matter. A third review author will resolve conflicts between independent review authors regarding inclusion/exclusion of articles. An excluded study list will record articles that did not meet inclusion criteria, but that may provide information of interest to readers.

### Data collection

We will extract data of interest from all included studies using revised versions of a previously piloted data extraction form. All review screening, bias assessment, and data extraction will be managed on the Covidence review management software. Specifically, we will extract variables outlined in Table 2 [28].

**Table 2:** Data extraction variables

Variable	Additional details
Study inclusion/exclusion	If excluded, rationale provided
Design and setting	
Dates	Study duration
*N*, number of groups, and dropout rates	
Participant demographics	This will include baseline cognitive status and activity or fitness level, diagnosis, disease severity, co-morbidities, Apo-E status, age, sex, nationality and/or ethnicity, socioeconomic status, and education
Included caregiver or caretaker	
Recruitment approach	
Adherence reported according to how intervention was recorded	For example, supervised, unsupervised, objective monitors, etc.
Follow-up assessment	
Outcomes	To include cognitive, emotional, functional, motor, physiological, social, or quality of life
Reported challenges and conflicts	
Bias assessment variables	To include randomization method, randomization concealment, blinding, method of reporting outcomes, missing information, and other relevant variables
Outcome description	To include scale range; minimal detectable change; and/or minimal clinically important difference; incidence of dementia; time to conversion; changes in health, cognitive, or quality of life variables; and changes in dementia related biomarkers
Other outcome factors	To include time to follow-up in study methodology, scale range, minimal detectable change, and/or minimal clinically important difference
Results: included participant group sizes, summary of results	For example, means, SD, and statistical changes
Physical activity/exercise treatment prescription data	
Physical activity/exercise modality	For example, type, group, peer/coach, and additional motivators
Treatment minutes/session	
Treatment duration	Total number of weeks
Supervised or unsupervised treatment paradigm	
Harm or side effects	Drug or secondary health condition interaction

### Risk of bias in individual studies

We will use the Risk of Bias 2 Tool (RoB 2) [29] to assess the risk of bias in all included articles at the study level. At least two review authors will assess risk of bias across the five RoB 2 domains, specifically bias related to randomization, deviation from intervention, missing data, measurement, and result selection [29]. A third review author will resolve any conflicts regarding risk of bias considerations. Finally, review authors will provide a rationale for all choices regarding risk of bias. A stratified analysis will be used to assess and report the levels of bias identified across included studies.

### Data synthesis

#### Qualitative synthesis

Abstracted and synthesized information will be organized in a tabular fashion. Summary tables will be used to explore the primary and secondary outcomes of interest extracted from included studies. The narrative summary in the tables will include the following: author, year of publication, country where the study was conducted, study objectives, population studied, outcome measures used, risk of bias, and major findings (see Table 1).

#### Quantitative synthesis

If two or more of the included studies reported the effect of the exercise intervention on a primary or secondary outcome, we will pool the eligible studies using a meta-analysis approach. Due to the expected heterogeneity of the included studies, we will use the inverse-variance random-effect model.

Revman version 5 will be used to pool estimates of the effect sizes on primary and secondary outcomes. Summary of pooled estimates will be also presented graphically in terms of forest plots.

### Measures of treatment effect

For dichotomous outcomes, we will calculate the odds ratio with a 95% confidence interval. For continuous outcomes, we will calculate the mean difference with a 95% confidence interval. We will use the standardized mean difference with a 95% confidence interval if the included trials use different scales for a continuous outcome. Hazard ratio will measure time-to-conversion to dementia data. We will use intention-to-treat data, and, if not available, we will only use the reported completers’ data in the analysis. The between-study heterogeneity will be measured using the chi-squared test. For chi-squared values with *P* < 0.1, heterogeneity will be considered to be significantly high. The *I*^2^ will be used to assess the inconsistency between the pooled studies [30]. The heterogeneity is determined as low, medium, or high when the *I*^2^ test values are 25%, 50%, or 75%, respectively [30]. The *I*^2^ of < 70% will be considered to be acceptable for pooling the data across the studies [30]. Any comparison with high heterogeneity will be explored by a subgroup analysis.

### Subgroup analyses

If sufficient studies are available (3 or more studies), we will perform subgroup analyses based on characteristics thought to modify treatment effects or possible sources of heterogeneity between studies such as age, gender, educational level, comorbidity, patient groups, types of intervention, or types of study. Our a priori hypothesis will be that older, lower educational level, and more comorbidity subgroups may show less improvement in the primary and secondary outcomes. We will also stratify our results by additional study method variables as appropriate to include supervised (exercise observed by clinical or exercise professional) versus unsupervised protocols, high versus low risk of bias, measurement through fitness versus heart rate, quality of the cognitive assessment used, and adherence over 80%.

#### Sensitivity analysis

We will use sensitivity analyses to assess the appropriateness of including various modes of exercise, such as aerobic and resistance training intervention protocols. In addition, we will use sensitivity analyses to assess whether or not the inclusion of studies with missing outcome data of varying degrees meaningfully impacts the results of the meta-analysis.

#### Meta-bias and quality of evidence

For each comparison with 10 or more studies, we will visually assess publication bias and small-study effects using funnel plots (using study’s effect estimates for the primary outcomes against their standard errors). We will utilize GRADE approach to determine the quality of evidence of included articles, designating all articles to one of four levels of evidence according to their study and other variables which have the potential to impact the quality of evidence (such as indirect evidence, specific types of bias, and effect size) [28].

## Discussion

This review protocol builds upon a growing body of literature to critically assess high-quality evidence in order to determine the conclusive impact of exercise on cognitive function. Declines in cognitive function, including memory, visuospatial skills, language skills, and executive function are hallmark in AD [6]. These cognitive changes are often paired with declines in activities of daily living and the ability to participate in meaningful tasks and social relationships. To address these deficits, pharmacotherapies such as acetylcholinesterase inhibitors and a N-methyl-D-aspartate (NMDA) agonist [7] are typically prescribed to help compensate for the effects of the disease. The benefits of pharmacotherapy can, unfortunately, be paired with unwanted side effects. Furthermore, these medications do not inhibit the progression of AD. The stress and negative mental health outcomes experienced by AD caregivers [31–33] further emphasize the importance of identifying and implementing effectual interventions for this population. Improved cognitive ability, subsequently enabling improved functional capacity, may lessen the responsibilities and challenges that informal caregivers face as they support their loved one with AD. A holistic and client-centered approach to caring for an individual with AD must then be appropriately informed of the value of supplementing pharmacotherapy with non-pharmacological interventions such as exercise prescriptions.

In addition to the direct physiological benefits of exercise, the accessibility and socioeconomic considerations of this intervention approach warrant its further consideration. Structured physical activity, if appropriately designed, can accommodate individuals with AD across diverse lifestyles and contexts. The recent outbreak of COVID-19 has brought attention to the importance of health care that can be prescribed remotely and carried out in one’s home. Exercise meets both of these criteria and is a highly malleable intervention, feasibly modifiable for context and safety during periods of social isolation or distancing.

We anticipate the outcomes of this review will indicate that structured exercise yields statistically and clinically significant improvements in health and quality of life factors. The effects of specific exercise protocols on various cognitive domains may imply the importance of certain volumes and intensities of exercise to address specific deficits. The results of this review are intended to guide clinical prescription and uptake of exercise protocols among the AD population as a targeted and effective intervention approach. In addition, the results of this review will likely hold implications for the impact of exercise on cognitive outcomes in additional neurodegenerative and age-related conditions. Once the review and meta-analysis is complete, results will be disseminated through both scientific, peer-reviewed journal article(s) and conference presentation. Any amendments made to this protocol when conducting the study will be outlined in PROSPERO and reported in the final manuscript.

### Potential limitations

The potential limitations of this systematic review include lack of clear and transparent reporting within included studies (for example, inclusion or exclusion criteria; cognitive level among sample participants; lack of consideration for gender, race, and socioeconomic status). A second potential limitation is the failure to include underrepresented minorities within study sample populations. Dependent on the reporting practices of the included studies, we also anticipate the potential for high heterogeneity due to lack of methodological rigor, for example, lack of blinding, lack of well-trained exercise specialist personnel, and failure to report if other motivational and engagement approaches were used in conjunction with the exercise treatment (e.g., music, group socialization, technology-based support).

There are several operational issues that we anticipate for this study. Firstly, lack of common data elements across studies may prove challenging in data extraction processes. A second potential issue is the likelihood that selection bias toward a sample of individuals with a relatively high literacy and socioeconomic status will limit generalizability of results to the general AD population. Finally, lack of data sharing and open access within science practices may provide additional challenges to the processes of this study.

This review is designed to assess and provide clinically relevant information regarding the impact of exercise on cognitive outcomes for those living with AD. Low cost, non-pharmacological and non-invasive interventions, such as exercise, are crucial considering the aging population and growing prevalence of AD. Interventions that can be implemented early in the disease process and in a person’s individual community context have the potential to significantly impact public health. Therefore, it is imperative to rigorously scrutinize, then purposefully implement the most effective interventions to help mitigate physical and cognitive decline associated with a diagnosis of AD. In sum, we anticipate that this review will yield clinically and socially meaningful insight into the impact of exercise on cognitive function in the AD population.

## Supplementary Information

**Additional file 1.** PRISMA-P 2015 Checklist.

**Additional file 2.** Search Strategies.

## Data Availability

The data used in this study will be extracted from published literature identified through Ovid MEDLINE(R) and Epub Ahead of Print, In-Process & Other Non-Indexed Citations and Daily.

## Abbreviation

AD: Alzheimer’s disease
EIM: Exercise is Medicine
NMDA: N-methyl-D-aspartate
RoB 2: Risk of Bias 2 Tool
SD: Standard Deviation
