# Supplementary material for: Exercise interventions for older adults with Alzheimer’s disease: a systematic review and meta-analysis protocol
Source: Syst Rev. 2021 Jan 4;10:6. doi: 10.1186/s13643-020-01555-8 (PMC7779651; doi:10.1186/s13643-020-01555-8)
Supplement: Supplementary file 2 — Additional file 2. Search Strategies. [file 13643_2020_1555_MOESM2_ESM.docx]

Additional File 2. Search Strategies

1. Ovid MEDLINE(R) and Epub Ahead of Print, In-Process & Other Non-Indexed Citations and Daily <1946 to November 10, 2020>

| **#** | **Searches** |
| --- | --- |
| 1 | dementia/ or alzheimer disease/ or (Alzheimer or Alzheimer's or Alzheimers or Dement$).ti,ab,kw. or AD.ti,ab. |
| 2 | exp Exercise/ or exp Exercise Therapy/ or exp Exercise Movement Techniques/ or (active lifestyle or physical activit$ or exercis$).ti,ab,kw. |
| 3 | 1 and 2 |
| 4 | exp Cognition/ or Cognitive Dysfunction/ or cognition disorders/ or (cognit$ dysfunct$ or cognit$ impair$ or cognit$ declin$ or mild cognit$ impair$ or MCI).ti,ab,kw. |
| 5 | exp Neuropsychological Tests/ or (Mini-Mental State Exam$ or mini-mental state or MMSE or Montreal Cognitive Assessment$ or MoCA).ti,ab,kw. |
| 6 | 4 or 5 |
| 7 | 3 and 6 |
| 8 | exp Aged/ or (aged or elderly or "65 years" or senior citizen$ or older adult$ or geriatric$).ti,ab,kw. |
| 9 | 7 and 8 |
| 10 | randomized controlled trial/ or Randomized Controlled Trials as Topic/ or randomized controlled trial.pt. or double-blind method/ or random allocation/ or single-blind method/ or (random$ or placebo).ti,ab. |
| 11 | (random$ or placebo).ti,ab,kw. and clinic$ trial$1.tw,kw. |
| 12 | 10 or 11 |
| 13 | 9 and 12 |
| 14 | 13 not (Animals/ not (Animals/ and Humans/)) |

1. **Embase.com (1947 – present)**

| #1 | 'dementia'/de OR 'alzheimer disease'/de OR alzheimer:ti,ab,kw OR alzheimers:ti,ab,kw OR ad:ti,ab OR dement*:ti,ab,kw |
| --- | --- |
| #2 | 'exercise'/exp OR 'kinesiotherapy'/exp OR 'physical activity, capacity and performance'/de OR 'active lifestyle':ti,ab,kw OR 'physical activit*':ti,ab,kw OR exercis*:ti,ab,kw |
| #3 | #1 AND #2 |
| #4 | 'cognition'/exp OR 'cognitive defect'/de OR 'mild cognitive impairment'/de OR 'dementia assessment'/exp OR 'cognitive function test'/exp OR 'mini-mental state exam*':ti,ab,kw OR 'mini-mental state':ti,ab,kw OR mmse:ti,ab OR 'montreal cognitive assessment*':ti,ab,kw OR moca:ti,ab |
| #5 | #3 AND #4 |
| #6 | 'geriatrics'/de OR 'aged'/exp OR aged:ti,ab,kw OR elderly:ti,ab,kw OR '65 years':ti,ab,kw OR 'senior citizen*':ti,ab,kw OR 'older adult*':ti,ab,kw OR geriatric*:ti,ab,kw |
| #7 | #5 AND #6 |
| #8 | 'randomized controlled trial'/de OR 'placebo'/de OR 'double blind procedure'/exp OR 'single blind procedure'/exp OR ((((clinical NEXT/1 trial*):ti,ab,kw) OR 'clinical trial'/exp) AND (random*:ti,ab,kw OR placebo*:ti,ab,kw)) |
| #9 | #7 AND #8 |

**PSycINFO (Ebsco) (1734- 2021)**

| **#** | **Query** |
| --- | --- |
| S9 | S7 AND S8 |
| S8 | ( DE "Randomized Controlled Trials" OR DE "Randomized Clinical Trials" OR DE "Placebo" OR DE "Random Sampling" ) OR TI ( ( randomized controlled trial OR rct OR random* OR placebo ) OR AB ( randomized controlled trial OR rct OR random* OR placebo ) ) OR AB ( ( randomized controlled trial OR rct OR random* OR placebo ) OR AB ( randomized controlled trial OR rct OR random* OR placebo ) ) |
| S7 | S5 AND S6 |
| S6 | DE "Geriatrics" OR DE "Aging" OR TI ( aged OR elderly OR "65 years" OR senior citizen* OR older adult* OR geriatric* ) OR AB ( aged OR elderly OR "65 years" OR senior citizen* OR older adult* OR geriatric* ) |
| S5 | S3 AND S4 |
| S4 | TI ( Cognitive dysfunction* OR cognitive impair* OR cognition disorder* OR cognitive declin* OR "mild cognitive impairment" OR Mini-Mental State OR “Montreal Cognitive Assessment” ) OR AB ( mild cognitive impairment" OR Mini-Mental State OR “Montreal Cognitive Assessment” ) OR TI ( MMSE OR MCI OR MoCA ) OR ( DE "Cognition" OR DE "Cognitive Impairment") OR (DE "Cognitive Assessment")) OR (DE "Mini Mental State Examination") ) |
| S3 | S1 AND S2 |
| S2 | ( DE "Physical Activity" OR DE "Exercise" OR DE "Aerobic Exercise" OR DE "Weightlifting" OR DE "Yoga" ) OR TI ( physical activit* or exercis* ) OR AB ( physical activit* or exercis* ) |
| S1 | ( DE "Dementia" OR DE "Alzheimer's Disease" ) OR TI ( Alzheimer OR Alzheimer's OR Alzheimers OR Dement* ) OR AB ( Alzheimer OR Alzheimer's OR Alzheimers OR Dement* ) |
